# Editorial Comment to Needlestick Injury During Urological Surgery: A 15‐Year Review From a Multi‐Institutional Study in the Chugoku–Shikoku Japan Urological Consortium

**DOI:** 10.1111/iju.70273

**Published:** 2025-10-30

**Authors:** Sayuri Takahashi

**Affiliations:** ^1^ Department of Urology, the Institute of Medical Science The University of Tokyo Tokyo Japan

**Keywords:** needlestick injury, risk management, urological surgery

Risk management in medicine is necessary for the safety of patients and medical staff. To prevent clinical accidents, occurrence reports should be collected and scientifically analyzed to elucidate the causes and find out the solution. Needlestick injuries (NSIs) are one of the most recognized accidents in the clinical environment which have high risks of infectious diseases to healthcare workers.

Sasaki et al. [1] have reported the review of a multi‐institutional study of NSIs during urological surgery for 15 years. Authors analyzed the data of 63 590 urological surgeries from seven institutes in the Chugoku–Shikoku area of Japan. The overall incidence of NSIs was 0.06% (range, 0.01%–0.24%). There were differences between robot‐assisted/laparoscopic surgery and open surgery; the incidence of NSIs was 0.18% in robot‐assisted surgeries, 0.21% in laparoscopic surgeries, and 0.02% in other procedures. Furthermore, NSIs were significantly more common during wound closure particularly in robot‐assisted/laparoscopic surgery. As a cause, authors guess it increased inadvertent puncture that multiple surgeons manipulated needles simultaneously during wound closure with adjusting the needle position using their fingers. They also stated surgeons became less vigilant during wound closure after the completion of main surgery.

Sasaki et al. reported no significant association observed between NSIs and operation time of surgeon experience. On the other hand, Krainev et al. reported the NSI rate by physician residents was more than threefold higher than the NSI rate by physicians (2 NSIs/100 physicians per year), when compared to National Surveillance System for Healthcare Workers data [2]. They described the significant factor was that residents were in training. As an aspect of education for residents, promoting a culture of reporting is important. Attending physicians can discuss their own experiences with suffering from NSIs during their careers, describe the importance of reporting injuries as they occur, and assure physician residents that they will not be penalized for experiencing or reporting a NSIs [2]. Al Yaqoubi et al. insisted a nationwide NSIs prevention training module, incorporating scenario‐based education, mandatory reporting, and adoption of safety‐engineered devices, is strongly recommended to reduce occupational risks and protect healthcare workers [3].

An interplay of multiple workers handling sharps instruments including residents, surgeons, and nurses would be effective to improve the operating room environment, and activating the reporting system of occurrences is critical to prevent NSIs from the viewpoint of risk management.

## Author Contributions

**Sayuri Takahashi:** conceptualization, writing.

## Conflicts of Interest

The author declares no conflicts of interest.

## Linked Articles

This article is linked to https://doi.org/10.1111/iju.70242.
